# Combined Exercise Training and l-Glutamine Supplementation Enhances Both Humoral and Cellular Immune Responses after Influenza Virus Vaccination in Elderly Subjects

**DOI:** 10.3390/vaccines8040685

**Published:** 2020-11-16

**Authors:** Fernanda R. Monteiro, Tamaris Roseira, Jonatas B. Amaral, Vitória Paixão, Ewin B. Almeida, Roberta Foster, Adriane Sperandio, Marcelo Rossi, Gislene R. Amirato, Juliana S. Apostólico, Carlos A. F. Santos, Eduardo S. Felismino, Fabyano B. Leal, Luciano M. Thomazelli, Edison L. Durigon, Danielle B. L. Oliveira, Rodolfo P. Vieira, Juliana M. B. Santos, André L. L. Bachi

**Affiliations:** 1 Department of Otorhinolaryngology, ENT Lab, Federal University of Sao Paulo (UNIFESP), Sao Paulo CEP 04025-002, Brazil; fernanda.monteiro@soufamesp.com.br (F.R.M.); tamaris.pavao@soufamesp.com.br (T.R.); jbamaral@unifesp.br (J.B.A.); v.paixao@unifesp.br (V.P.); ewin.almeida@unifesp.br (E.B.A.); roberta.foster@timefamesp.com.br (R.F.); mrossi@dim.fm.usp.br (M.R.); prof.gislene@hotmail.com (G.R.A.); freitas.carlos@uol.com.br (C.A.F.S.); abachi@unifesp.br (A.L.L.B.); 2 Method Faculty of Sao Paulo (FAMESP), Sao Paulo CEP 04046-200, Brazil; ane.sperandio@gmail.com; 3 Department of Microbiology, Immunology and Parasitology, Federal University of Sao Paulo (UNIFESP/EPM), Sao Paulo CEP 04025-002, Brazil; juliana.apostolico@unifesp.br; 4 Post-graduation Program in Health Science, Santo Amaro University (UNISA), Sao Paulo CEP 04829300, Brazil; efelismino@estudante.unisa.br; 5 Institute of Biomedical Science of University of Sao Paulo (USP), Sao Paulo CEP 05508-900, Brazil; fabyo_leal@icb.usp.br (F.B.L.); lucmt@usp.br (L.M.T.); eldurigo@usp.br (E.L.D.); danibruna@gmail.com (D.B.L.O.); 6 Scientific Platform Pasteur USP, Sao Paulo CEP 05508-020, Brazil; 7 Hospital Israelita Albert Einstein, Sao Paulo CEP 05652-900, Brazil; 8 Brazilian Institute of Teaching and Research in Pulmonary and Exercise Immunology (IBEPIPE), Sao Jose dos Campos CEP 12245-520, Brazil; rodrelena@yahoo.com.br; 9 Post-graduation Program in Bioengineering and Biomedical Engineering, Universidade Brasil, Sao Paulo CEP 08230-030, Brazil; 10 Post-graduation Program in Science of Human and Rehabilitation, Federal University of Sao Paulo (UNIFESP), Santos CEP 11060-001, Brazil

**Keywords:** flu vaccine, amino acid, immunoglobulins, lymphocytes, exercise training

## Abstract

Background: Since aging affects the immune responses against vaccination, the present study evaluated the effects of L-glutamine (Gln) supplementation in the humoral and cellular immune responses in elderly subjects, practitioners or not, of physical exercise training. Methods: Eighty-four elderly people (aged 72.6 ± 6.1), non-practitioners (NP, *n* = 31), and practitioners of combined-exercise training (CET, *n* = 53) were submitted to Influenza virus vaccination and supplemented with Gln (0.3 g/kg of weight + 10 g of maltodextrin, groups: NP-Gln (*n* = 14), and CET-Gln (*n* = 26)), or placebo (10 g of maltodextrin, groups: NP-PL (*n* = 17), and CET-PL (*n* = 27)). Blood samples were collected pre (baseline) and 30 days post-vaccination and supplementation. Results: Comparing with the baseline values, whereas the NP-Gln and CET-PL groups showed higher specific-IgM levels, the CET-Gln group showed higher specific-IgM and IgA levels post-vaccination. The titer rate of hemagglutination inhibition was higher in the CET-Gln, NP-PL, and NP-Gln groups post-vaccination than baseline values. The absolute number of naive and effector CD4+ T cells was higher especially in the NP-Gln and CET-Gln groups, whilst activated CD4+ T cells were higher in CET subgroups post-vaccination. Conclusion: Our results showed that both l-glutamine supplementation and combined-exercise training can improve the immune responses to the Influenza virus vaccine in elderly subjects.

## 1. Introduction

Based on the data regarding the pace of population aging, it is estimated that by 2050 the proportion of the world’s population over 60 years of age will increase by 22%. Furthermore, it is noteworthy to mention that it is also estimated that 80% of these elder people will live in developing countries [1]. Therefore, it is of the utmost importance to promote actions in order to achieve healthy aging.

Aging is a natural and multifactorial phenomenon, in which its corollary aspect is associated with inevitable changes related to age that elicits a gradual decline in several systems, with highlights to the immune system [2]. The prominent alterations in the immune system observed during aging are named as immunosenescence [3]. Among some hallmarks of immunosenescence, there is no doubt that the profound immunological changes with advancing age affect both the immune cells production and its action in the periphery and, consequently, the older individual becomes unable to defend itself from pathogens or even generate an efficient immune response against some challenges, mainly the vaccination [4].

In this respect, several studies have demonstrated reduced specific antibodies responses against the influenza vaccine in elderly people [5], showing that immune cell actions are severely compromised during the immunosenescence [6]. The best proof for such findings is that it was reported a significant decline in the number of T cells, particularly naive T cells, associated with an increase of memory T cells [7]. Therefore, it is broadly accepted that these aspects can putatively disturb the immune responses against the new challenges imposed by the vaccination [8].

In order to minimize the deleterious effects of immunosenescence, it has been reported that the regular practice of physical exercise can be considered a powerful non-pharmacological intervention. For instance, our group previously demonstrated that elderly people practitioners of a regular program of combined-exercise training presented higher specific antibodies levels (both IgM and IgG) against the influenza vaccine as compared to a group of sedentary elderly people [9].

Beyond the physical exercise, it is also proposed that some dietary interventions, such as supplementation with protein or amino acids, could be helpful to mitigate the immunosenescence [10]. In this context, the supplementation effect with L-glutamine (Gln), a non-essential amino acid, showed the capacity to help not only the proliferation but also the cytokine production by the lymphocytes. These positive effects of Gln in the lymphocytes can be attributed mainly to the fact that this amino acid is largely used as the main energy source, which can lead to increased glycogen production. In addition, Gln also showed anticatabolic effects and improve water and electrolytes absorption, which in association with its “energetic” support can putatively influence the better “clonal expansion” capacity and in the immune response [11].

Gln is the most abundant amino acids both in plasma and in the skeletal muscles, and, in combination with physical exercise at moderate-intensity by elderly people, can minimize some deleterious effects of aging in different aspects, such as muscle strength recovery and soreness [12], reestablishing the redox and the inflammatory balance [13].

Although it has clear that Gln present functional properties, especially in the immune and musculoskeletal systems, the effects of Gln supplementation in the specific immune response against the Influenza virus vaccine in a population of exercised elderly individuals has never been evaluated. Therefore, in this study, we investigated the specific antibodies levels (IgA, IgM, and IgG) in response to Influenza virus vaccination, as well as the number and activation of T CD4+ cells (both naive and effector) in elderly people, participated or not of a regular program of combined-exercise training, and supplemented or not with Gln.

## 2. Materials and Methods

### 2.1. Subjects and Study Design

**Figure 1:**
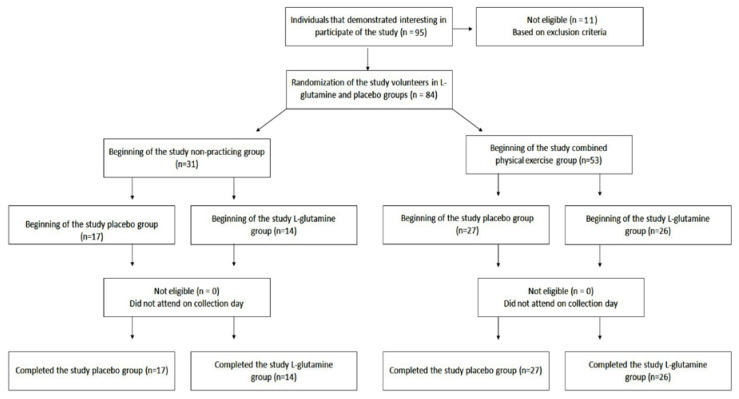
Flow diagram of the study.

Eighty-four (*n* = 84) elderly people aged between 60 and 85 years (aged 72.6 ± 6.1), both men (*n* = 19) and women (*n* = 65) were enrolled in the study. All the information concerning the recruitment, selection, and participation of the elderly population are presented in Figure 1. The supplement randomization is described below in the Section 2.2. Similarly, to previously reported [13], all volunteers were recruited from the Primary Health Care Program belonging to the Geriatrics and Gerontology Discipline of the Federal University of São Paulo (UNIFESP). Volunteers were separated into two groups: non-practitioners group (NP, *n* = 31; men = 8 and women = 23) and combined-exercise training group (CET, *n* = 53; men = 11 and women = 42). All participants signed the informed consent form previously approved by the Ethics Committee of the Federal University of São Paulo (approval number 3.623.247) and CAEE (18170619.3.0000.5505). The study was in agreement with the Ethical Standards of exercise practice [14], as well as all experiments were performed per the Declaration of Helsinki.

Anthropometric characteristics (weight, height, and body mass index (BMI)) were assessed. In addition, the Food Frequency Questionnaire (FFQ) was used to evaluate the dietary consumption of protein, as well as the occurrence of amino acid supplementation. None of the volunteers participating in this study had neoplasms, chronic infections, neurological, renal and/or liver diseases, thrombosis and cardiovascular diseases, type I diabetes mellitus, or other illnesses that prevent the practice of physical exercises. The same physician at the Geriatrics and Gerontology Discipline of the Federal University of São Paulo (UNIFESP) performed the clinical and physical examinations.

### 2.2. Combined-Exercise Training (CET)

During the evaluated period, volunteers from the combined-exercise training (CET) group, supplemented with Gln or placebo, maintained their usual training routine consisting of a prescribed exercise program for 60–75 min per session, 3 times per week, on alternate days, for 30 days. Emphasizing that the volunteers have been participating in this program for at least 12 months and were supervised by the same physical education professional.

As described by Almeida et al. [13], the combined exercise program was structured with aerobics and strength exercises. Aerobic training was performed between 60 to 75% of the maximal cardiac frequency (estimated by the equation (208 − 0.7 × age)). The strength training was performed also in a moderate intensity, between 50 to 60% of a maximal strength repetition, according American College of Sports Medicine (ACSM) [15] and training session intensity was assessed by the Borg Scale of Perceived Exertion, the protocol was applied 2 to 3 times per week and was preconized 5 to 10 different exercises, involving at least two muscle groups per session.

### 2.3. Non-Practicing Group

The recruitment process of volunteers from the non-practicing group was carried out by a co-author (CASF), the physician responsible for the Primary Health Care Program belonging to the Geriatrics and Gerontology Discipline of the Federal University of São Paulo (UNIFESP), through the database of this Discipline. Following the selection, all those volunteers were invited to participate in the study and, during the clinical and physical examinations, all of them were inquired in relation to daily routine and whether they were engaged in a regular exercise training program.

Moreover, in order to assess the level of physical activity in the volunteer groups, it was used the International Physical Activity Questionnaire (IPAQ) [16], validated for the Brazilian population [17]. By IPAQ it is possible to estimate weekly time spent on physical activities of moderate and strong intensity as well as sitting state, in different contexts of daily life. The results obtained in the IPAQ can be presented in METs (metabolic equivalents) or minutes per week. Based on the WHO recommendations, the cut-off associated with adequate physical activity for elderly people is 150 min per week of physical activity. Therefore, elderly subjects presenting values more than 150 min per week of physical activity are considered physically active, whereas elderly people presenting values lower than 150 min per week are considered sedentary subjects [18].

### 2.4. l-Glutamine or Placebo Supplementation

Volunteers of NP and CET groups were randomly separated into 2 subgroups: NP-placebo (NP-PL, *n* = 17); NP-l-glutamine (NP-Gln, *n* = 14); CET-placebo (CET-PL, *n* = 27); and CET l-glutamine (CET-Gln, *n* = 26). It is noteworthy to clarify that the randomization of the supplement was performed following this process: first of all, one co-investigator (JBA) created a list using computer-generated random numbers in advance. After that, each participant recruited received a code number in order of recruitment. Finally, the supplementation (PL or Gln) was provided using the list, and the code number attributed to each volunteer. 

The groups supplemented with l-glutamine ingested 0.3 g/kg of weight/day of l-glutamine (Tongliao Meihua Biological Sci Tech Co. Ltd., Tongliao, China) added with 10 g/day of maltodextrin (PR Netto Indústria e Comércio de Alimentos Ltd.a., Sao Paulo, Brazil). It is worthy to clarify that the dose of L-glutamine used here followed that described in the study of Legault et al. [12,13]. The subgroups supplemented with placebo ingested only 10 g/day of maltodextrin. All the volunteers were oriented to dilute 1 sachet of the supplement (L-glutamine or placebo) in 250 mL of water and ingest the solution immediately, for 30 consecutive days. In addition, all the participants were oriented to interrupt immediately the ingestion of any type of supplementation if any gastrointestinal disturbance was perceived.

### 2.5. Influenza Vaccine

The volunteer groups received the seasonal Influenza virus vaccine available in 2019. This trivalent vaccine was composed of two types of influenza A virus (A/Michigan/45/2015 (H1N1), and A/Switzerland/8060/2017(H3N2)) and one type of influenza B virus (B/Colorado/06/2017). The vaccine was administered intramuscularly.

### 2.6. Collection of the Samples

Fasting blood samples were collected in two different time-points: before (pre) and 30 days after (post) of vaccination and supplementation. It is important to mention that the volunteers in the CET group were instructed to perform the last physical exercise session 24 h before blood sampling collection. 

Blood samples were collected in tubes containing EDTA in order to obtain the peripheral blood mononuclear cells, which were used in immunophenotyping analysis. Another blood sample was collected in tubes without any anticoagulant compound in order to obtain sera aliquots, which were used to determine the specific IgA, IgM, and IgG antibodies levels against the Influenza, and its neutralizing capacity by hemagglutination-inhibition (HI) assay.

### 2.7. Immunophenotypic Profile

**Figure 2:**
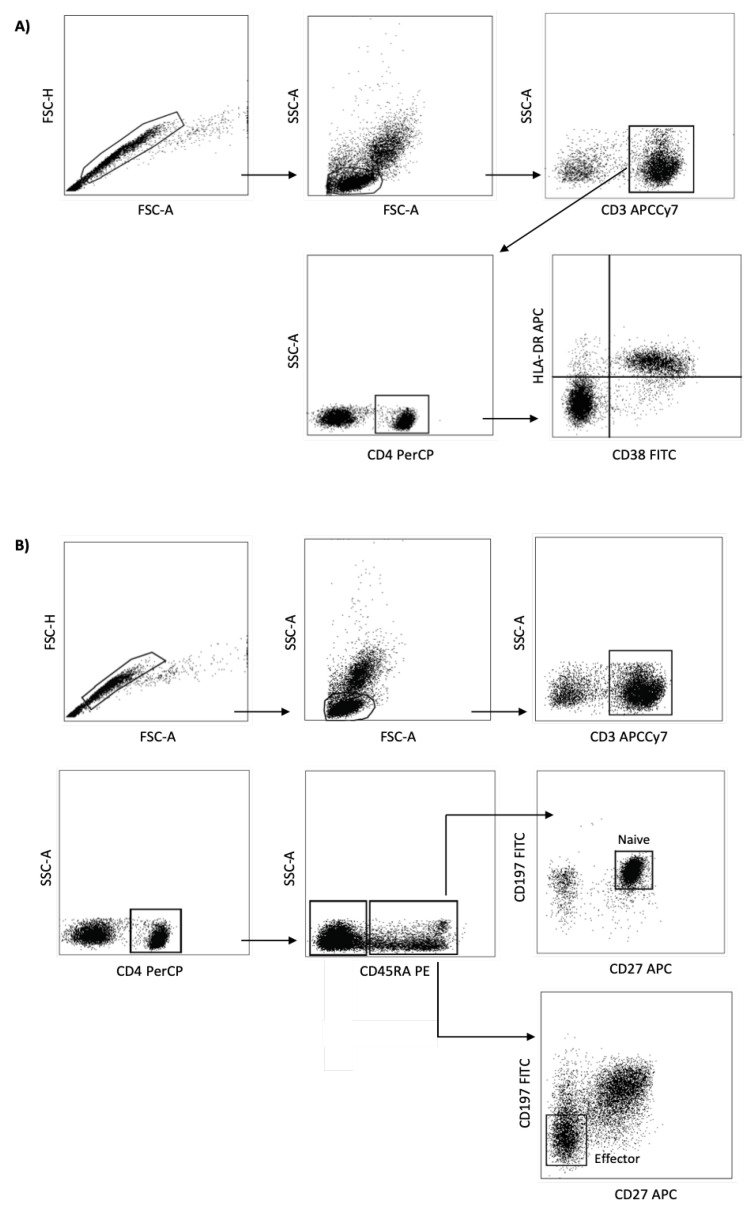
Flow cytometer analysis of cellular activation, CD4+ T cells naive and effector memory. Representative dot plots of a flow cytometry panel used for the detection of CD4+ T cells activated (**A**) and CD4+ T cells naive and effector (**B**). PBMCs were stained with Abs recognizing CD3, CD4, CD8, HLADR, CD38, CD45RA, CD27, and CD197 and were analyzed by flow cytometry, gating as indicated.

Briefly, after blood sampling collection, the blood was mixed 1:1 with phosphate-saline buffer (PBS 1×, pH = 7.3) and the peripheral blood mononuclear cells were obtained using Ficoll-Hypaque (GE Healthcare Bio-Sciences AB, Uppsala, Sweden). To evaluate the activation profile of CD4+ T cells, 1 × 10^6^ cells were used for immunostaining using the monoclonal antibodies for FACS assays: anti-CD3 APCCy7 (clone SK7), anti-CD4 PerCP (clone RPA-T4), anti-CD38 FITC (clone HIT2), and anti-HLA-DR APC (clone L243). T cell activation were determined as CD3+CD4+HLA-DR+CD38+ and to evaluate the absolute number of naïve and effector CD4+ T cells using anti-CD3 APCCy7 (clone SK7), anti-CD4 PerCP (clone RPA-T4), anti-CD27 APC (clone O323), anti-CD45RA PE (clone HI100), and anti-CD197 FITC (CCR7, clone G043H7). CD4+ T cell naïve and effector were determined as CD3+CD4+CD45RA+CD27+CD197+ and CD3+CD4+CD45RA+CD27-CD197-, respectively. The description of the panel used in this study is shown in Table 1 and the analyses strategies showed in Figure 2.

The cells were centrifuged at 1700 rpm for 5 min and transferred into 96 V bottom well plates (Nalge Nunc, Rochester, NY, USA) in 100 uL of FACS Buffer (PBS supplemented with 0.5% BSA and 2 mM EDTA) containing the antibodies (all from BD Biosciences, San Jose, CA, USA). Cells were incubated at 4 °C in the dark for 30 min, washed twice, and resuspended in 100 µL of fixation buffer (1% paraformaldehyde in PBS, (pH 7.4). Five hundred thousand events were acquired on a FACSCanto II flow cytometer (BD Biosciences) and then analyzed using FlowJo software (version 10.2, BD Biosciences, San Carlo, CA, USA). In order to obtain robust and reliable results concerning the phenotypical differentiation of the CD4+ T cell, an analysis strategy was carried out known as FMO (fluorescence minus one), which is widely used to identify more precisely where to select the cell population gates of interest when multiple fluorochromes are used in a given panel.

### 2.8. Determination of Specific Antibodies (IgA, IgM, IgG) for the Flu Vaccine (Influenza Virus)

The serum concentration of the specific antibodies (IgA, IgM, and IgG) against the Influenza virus vaccine was measured by ELISA “in house” test following the previously described by our group in Bachi et al. [9]. In all ELISA tests, the sera were diluted 1:4000, for IgA and IgM measurement, and 1:10,000, for IgG measurement, in PBS + 0.1% Tween (PBS-T) containing 0.25% BSA (PBS-T-BSA) and the secondary antibodies (peroxidase-conjugated anti-human) IgA, IgM and IgG (Sigma, St. Louis, MO, USA) were diluted 1:4000 in PBS-T-BSA. Absorbance was read at 450 nm on a microplate reader (Multiskan Sky Microplate Spectrophotometer, ThermoFisher, Waltham, MA, USA).

### 2.9. Hemagglutination-Inhibition (HI) Assay 

The hemagglutination-inhibition (HI) assay was performed using a viral solution contained influenza A (IA) H1N1 virus, which was grown in cell culture (MDCK) and cryopreserved in HEPES on −80 °C ultrafreezer. The concentration of IA H1N1 virus was adjusted to 4 hemagglutination units (HAU) per 50 μL in PBS before use. The same volume (1:1 *v*/*v*) of chicken red blood cells (CRBCs) and Alsever (LGC Bio) were used for assays performed up to 7 days post-bleed date. CRBCs were washed and resuspended to a final concentration of 1 in PBS (1×) prior use. The HI assay was carried out using 96-well round-bottom plates. Briefly, 50 μL of PBS was added to all wells, including wells in column 12, this column served as the erythrocytes controls. 50 μL of individual clinical test sera was added to each row of column 1 (A–H). All sera were serially diluted in twofold increments through column 1 to 11, resulting in dilutions from 1:2 to 1:2048. After serial dilution, 50 μL (4 HAU) of the diluted IA H1N1 virus was added to each well except for the erythrocytes control wells (rows A to H in column 12). Plates were then incubated for at least 30 min at room temperature. After incubation, 50 μL of 1% CRBCs was added to every well on all plates. Plates were tapped to ensure mixing, covered, and incubated for 30 min at room temperature. Plates were then tilted and wells observed for agglutination. Non agglutinating cells were defined by a button of cells at the very bottom of the well, agglutinating cells were defined as a diffuse pattern of settling on the well bottom with no running or streaking to the edge of the well when the plate was tipped. The HI titer of the serum sample was determined to be the inverse of the last dilution where cells were not agglutinated.

### 2.10. Statistical Analysis

Initially, we compared the continuous and semicontinuous data with the Gauss curve and the normality for each was determined by the Kolmogorov-Smirnov test. In addition, the homogeneity of variance was evaluated by the Levene test.

Parametric variables were presented as mean and standard error. The Student *t*-test was used to verify the occurrence of significant differences in the anthropometric data between the volunteer groups (NP × CET).

Non-parametric variables were presented as median and interquartile range. The Wilcoxon-signed rank test was used in the intragroup analysis to determine whether the differences between pre-vaccination values (PRE) and 30 days post-vaccination values (POST) were significant in each group supplemented with l-glutamine or placebo. The Kruskal–Wallis test with the Muller–Dunn post-test was used in the intergroup analysis to identify significant differences between the two groups (NP × CET), supplemented with l-glutamine or placebo.

The α risk considered in this study was set at 5% (*p* < 0.05).

## 3. Results

Table 2 shows anthropometric characteristics and physical activity levels of the volunteers from the NP and CET groups, both supplemented with l-glutamine (NP-Gln and CET-Gln) or placebo (NP-PL and CET-PL). It is possible to observe that both height and weight in the elderly volunteers in the NP-Gln subgroup were higher than the values found in the other subgroups. In relation to body mass index (BMI), the NP subgroups presented higher BMI than CET subgroups, respectively. Regarding the data obtained by IPAQ, the NP subgroups showed not only lower physical activity levels but also higher sitting time than the values found in the CET subgroups, respectively. Although these last significant differences were expected, it is very important to mention that the physical activity levels found in the NP subgroups were above the baseline of sedentarism (150 min of physical exercise per week, min/w), which allows us to classify, these volunteers, with active elderly subjects.

### 3.1. Combined Exercise Training and l-Glutamine Supplementation Improves the Specific Antibodies Response for Influenza Vaccine

In order to respond to our aim, first we carried out the evaluation of specific-polyclonal antibodies in response to the influenza virus vaccine in the volunteer CET and NP groups supplemented with Gln or placebo participating in this study.

**Figure 3:**
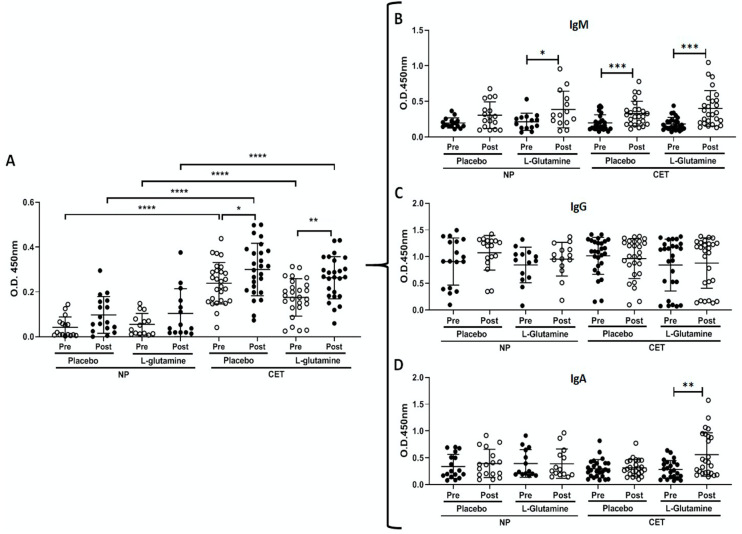
Total concentration of polyclonal antibodies (**A**) [IgA (**B**), IgM (**C**) and IgG (**D**)] specific for influenza vaccine in the groups of elderly non-practitioners (NP) and practitioners of combined-exercise training (CET), supplemented with L-glutamine or placebo. The concentrations were determined in serum samples obtained before (Pre) and after 30 days (Post) of vaccination and supplementation. Significance level of * *p* < 0.05, ** *p* < 0.01, *** *p* < 0.001, **** *p* < 0.0001.

As shown in Figure 3A, the intergroup analysis showed that the CET subgroups presented higher levels of the specific-polyclonal antibodies against the influenza virus vaccine before the vaccination than the levels observed in the NP subgroups. In a similar way, 30 days after vaccination and supplementation the significant differences between these subgroups were maintained. Concerning the intragroup analysis, both CET subgroups showed increased levels of the specific-polyclonal antibodies 30 days after vaccination and supplementation as compared to the values found before. However, it is noteworthy to point out that the statistical difference found in the CET-Gln subgroup was more significant (*p* = 0.008) than the value observed in the CET-PL subgroup (*p* = 0.04).

Based on the observation that the specific-polyclonal antibodies against the influenza virus vaccine in the CET subgroups increased post-vaccination and supplementation, we carried out a second round of antibody analysis, but in this time evaluating the serum levels of three different immunoglobulin isotypes (IgM, IgG, and IgA). The results regarding these analyses are shown in Figure 3B–D. 

In Figure 3B is showed that the specific-IgM levels in response to the influenza virus vaccine were higher post-vaccination and supplementation in the NP-Gln (*p* < 0.05), CET-PL (*p* < 0.001), and CET-Gln (*p* < 0.001) subgroups than the levels observed before (pre). In relation to the specific-IgG levels in response to the influenza virus vaccine (Figure 3C), no differences were found. Figure 3D shows that the CET-Gln subgroup presented increased (*p* < 0.01) specific-IgA levels in response to the influenza virus vaccine post-vaccination and supplementation as compared to the values before (pre).

### 3.2. l-Glutamine Supplementation Improved the Neutralizing Antibody Capacity

**Figure 4:**
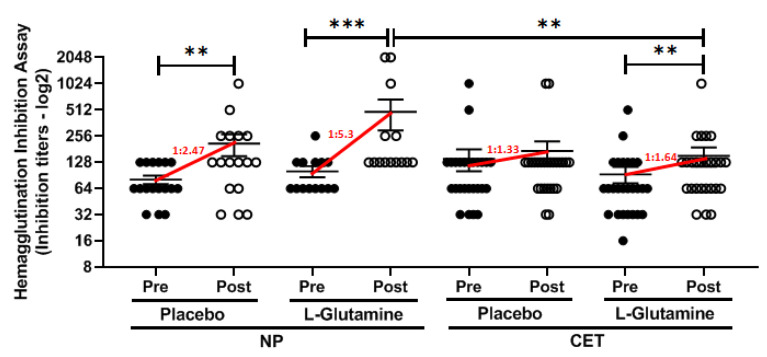
Hemagglutination inhibition (HI) assay antibody titers (log2) against the H1N1 virus in the groups of elderly non-practitioners (NP) and practitioners of combined-exercise training (CET), supplemented with L-glutamine or placebo. The antibody titers were determined in serum samples obtained before (Pre) and after 30 days (Post) of vaccination and supplementation. Red lines indicate the antibody titer increase rate. Significance level of ** *p* < 0.01, *** *p* < 0.001.

In order to evaluate the specific-antibody capacity to neutralize the H1N1 virus, it was performed the hemagglutination inhibition assay. As shown in Figure 4, both CET subgroups showed a small titer increase rate (CET-PL = 1:1.33 and CET-Gln = 1:1.64), whereas in the NP-PL increase of the titer rate was 1:2.47 and in the NP-Gln was found the higher increase of the titer rate (1:5.3). According to the statistical analysis, significant differences were observed in the titer rate in the subgroups CET-Gln (*p* = 0.004), NP-PL (*p* = 0.002), and NP-Gln (*p* < 0.001). In addition, the titer rate in the NP-Gln subgroup was also statistically different from the values found in the CET-Gln subgroup (*p* = 0.002). It is of utmost importance to mention that the statistical result related to the titer rate found in the CET-PL subgroup was *p* = 0.053.

### 3.3. l-Glutamine Supplementation Increases the Absolute Number of Effector CD4+ T Cells, Whereas the CET Increase the Activated CD4+ T Cells

**Figure 5:**
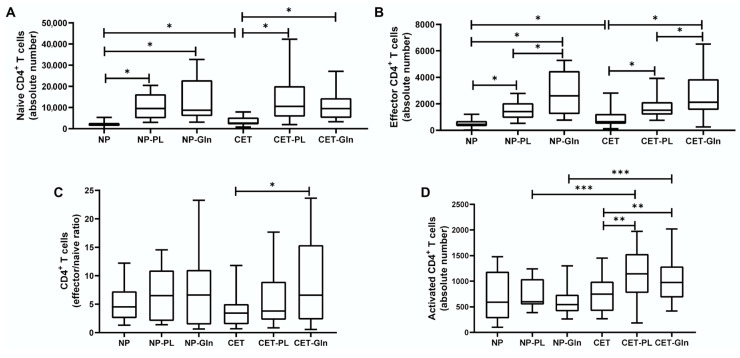
Immunophenotyping and activation of TCD4+ lymphocyte. (**A**) absolute number of naive CD4+ T cells; (**B**) absolute number of effector CD4+ T cells; (**C**) ratio between the absolute number of naive CD4+ T cells by effector CD4+ T cells; (**D**) absolute number of activated CD4+ T cells. Significance level of * *p* < 0.05, ** *p* < 0.01, *** *p* < 0.001.

Beyond the evaluations regarding the humoral response focused on the specific-antibody response, we also performed the analysis of the cellular response, emphasizing the immunophenotyping and activation of CD4+ T cells (Figure 5). 

Concerning the data of absolute number of naive CD4+ T cells shown in Figure 5A, before the supplementation, the CET subgroups presented higher number of this subtype of lymphocyte as compared to the NP subgroups. Post-vaccination and supplementation, all subgroups showed a significant increase in this number of CD4+ T cells in relation to the values before (pre). 

In relation to the evaluation of the absolute number of effector CD4+ T cells (Figure 5B), similarly to described above, before the supplementation, the CET subgroups also presented a higher number of this lymphocyte as compared to the NP subgroups, as well as post-vaccination and supplementation all subgroups showed a significant increase in comparison to the values before (pre). However, it is worth mentioning that the values observed in the subgroups supplemented with L-glutamine (NP-Gln and CET-Gln) were also statistically higher than the numbers found in the subgroups supplemented with placebo (NP- PL and CET-PL).

Figure 5C shows the results obtained in the ratio between the absolute number of effector CD4+ T cells by naive CD4+ T cells. It was verified that the CET-Gln subgroup presented increased ratio as compared to the values before (pre).

Regarding Figure 5D, whereas pre-vaccination and supplementation the absolute number of activated CD4+ T cells were not different between NP and CET groups, post-vaccination and supplementation the CET subgroups showed a higher percentage than the NP subgroups. In addition, the percentage found in the CET subgroups was also statistically increased in comparison to the values before (pre).

## 4. Discussion

**Figure 6:**
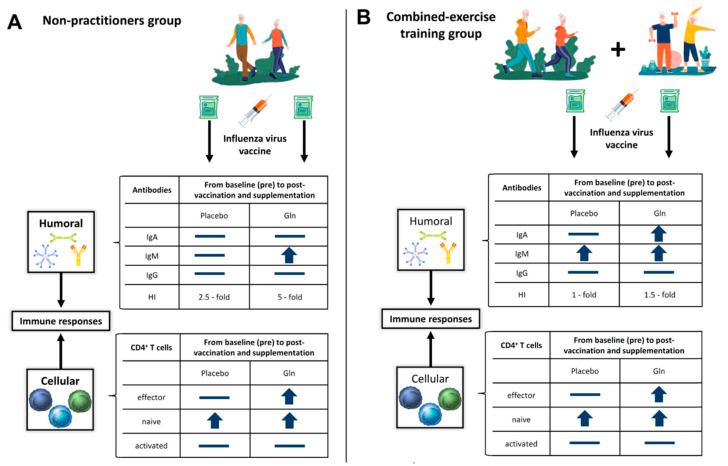
Representative illustration of the main findings of the study. All volunteer groups (Non-Practitioners (**A**), and Combined-exercise training (**B**), were submitted to blood sampling before and 30 days after Influenza virus vaccination and supplementation with placebo (PL) or l-glutamine (Gln). The arrows indicate that the immunological parameter evaluated increased post-vaccination and supplementation as compared to baseline values, whereas the symbol indicates that the values for the immunological parameter evaluated unchanged during the study. Abbreviation: CD = cluster differentiation; IgA = immunoglobulin A; IgM = immunoglobulin M; IgG = immunoglobulin G; HI = hemagglutination-inhibition.

Our results showed, for the first time, that the practice of regular CET by elderly subjects when associated with Gln supplementation was able to induce an increase in the levels of the specific-IgM and -IgA antibodies against the influenza vaccine. Interestingly, the Gln supplementation was able to improve the specific-IgM response to vaccination in the elderly subjects’ non-practitioners of combined exercise training program. Regarding the specific-IgG response to vaccination in elderly groups participating in this study, no differences were found. Beyond these evaluations, it was also verified that all subgroups showed increased the titer of hemagglutination inhibition by antibodies after vaccination, in special in the NP subgroup supplemented with Gln. In addition, we also found that the absolute number of naive CD4+ T cells increased after vaccination regardless of Gln supplementation, whereas the absolute number of effector CD4+ T cells increased especially in the subgroups supplemented with Gln. Finally, a higher number of activated CD4+ T cells were also only observed in the CET subgroups post-vaccination (Figure 6).

It is well-known that the immunosenescence can disturb the capacity of elderly subjects to respond against the influenza vaccine, especially due to the alteration in the number of naive and memory T lymphocytes [19]. Concerning the regular practice of physical exercise, especially the CET, it has been demonstrated that it can benefit the elderly population in different ways. For instance, it has been demonstrated that CET can putatively reduce the incidence of upper airway infection when compared to the values observed in sedentary individuals [20]. It should be emphasized that elderly subjects who presenting an active lifestyle, with prominent engagement in long-standing physical exercise programs show a better immune response against influenza vaccination [9,21,22], particularly by the reduction of senescent T lymphocytes and also by increase the number of naive and effector T lymphocytes [23]. 

These remarkable effects of physical exercise on the T lymphocytes were reported in studies that showed that after a session of physical exercise there was an increase in the induction of apoptosis in CD4+ T cells, especially those with senescent phenotype, in the elderly subjects. It is notable to mention that this evidence led several authors to postulate that the apoptosis of these cells could generate a “space” and, by a physiological turnover, this space could be filled by new cells, mainly by naive and effector CD4+ T cells [24,25,26]. Our results corroborate these findings since the CET group showed an increased absolute number of naive and effector CD4+ T cells as compared to the NP group.

Our observation that the NP subgroups also were able to increase both naive and effector CD4+ T cells allows us to suggest that an active lifestyle can be able to mitigate one of the hallmarks attributed to immunosenescence. According to the literature, even though the ability to develop naive CD4+ T cells is limited in elderly individuals, the increase in the number of these cells in physically active individuals may be related to the homeostatic proliferation of pre-existing naive CD4+ T cells [27]. Therefore, the increase in the number of naive CD4+ T cells in association with the maintenance of the homeostatic proliferation capacity of pre-existing lymphocytes in the periphery may have favored the significant increase in the number of effector CD4+ T cells in both groups of elderly volunteers participating in this study. However, it is important to mention that the L-glutamine supplementation was able to improve the number of effector CD4+ T cells in our study.

In a different way of the data found for naive and effector CD4+ T cells, only the CET subgroups showed a significant increase in the number of activated CD4+ T cells 30 days post-vaccination. In agreement with Stervbo et al. [28], T lymphocytes obtained from young individuals showed fast activation and proliferation in response to the Influenza vaccine, whereas a reduced number of activated and proliferating T lymphocytes were observed post-vaccination in the elderly. Moreover, the kinetics of changes in activated T lymphocytes was more evident in young individuals, while the corresponding changes in the elderly occurred more slowly. Based on these data, the increased number of activated CD4+ T cells in the CET subgroups post-vaccination reinforces the potential effect of physical exercise in modulating the activation of these cells in the elderly people, which was not observed in the NP subgroups.

Corroborating the benefits of the regular practice of combined exercise training on the immune response against vaccination in this study, the CET group showed increased IgM levels post-vaccination as compared to the values before, regardless of supplementation. This find is in agreement with our former observation in which an exercised elderly group presented higher specific-IgM levels after the Influenza vaccination when compared to the values found prior to the vaccination and also to the sedentary group [9]. Although the results regarding the specific-IgG levels for the Influenza vaccine found in this study were unexpected and were different from our previous reports [9], there are a handful of reports in the literature showing that the annual elderly vaccination for Influenza virus can impact this population in a negative way, for instance, leading them to present similar systemic IgG levels prior and post-vaccination [29], as observed in this study.

It is noteworthy to mention that, beyond the evaluation of combined exercise training, in this study, it was also assessed the effect of Gln supplementation in the immune response of elderly subjects submitted to Influenza vaccination. As previously mentioned, Gln is one of the most abundant amino acids in plasma and skeletal muscles and, mainly, is considered as an essential fuel source for immune cells [12]. According to Almeida et al. [13], combining Gln supplementation with moderate exercise training was able to mitigate some deleterious effects of aging in elderly subjects. By the way, our findings also showed a remarkable effect of Gln supplementation on immunosenescence, mainly in terms of the specific antibody response (IgM and IgA) against Influenza vaccination and the absolute number of effector CD4+ T cells.

Concerning the specific-IgM for the Influenza vaccine, both subgroups supplemented with L-glutamine (NP-Gln and CET-Gln) presented higher levels of this antibody as compared to the prior values. Although some studies have shown an increase in IgM levels in individuals supplemented with Gln [30,31,32], as far as we can verify, this is the first study that demonstrates an increase in specific-IgM levels post-vaccination for the Influenza virus in an elderly population. In addition, it is remarkable to cite that the same two subgroups also presented an increased absolute number of effector CD4+ T cells in comparison to the subgroups supplemented with placebo and the prior numbers. So, the elevation of the absolute number of these immune cells could positively impact the better humoral immunological response observed in these volunteer subgroups supplemented with Gln.

Interestingly, and as one of the novelties of this study, it was found that the specific-IgA levels for the Influenza vaccine were higher both in serum and in saliva when the Gln supplementation was associated with regular practice of combined exercise training. Although there is data in the literature reports showing that exercise training can increase the total secretory IgA levels in saliva, including in elderly subjects [21,33,34], there are few studies showing serum elevation of this antibody in exercised individuals [35]. Based on the data showed by Klentrou et al. [33] the increase of total secretory IgA in saliva by a volunteer group submitted to a program of exercise training was negatively correlated with the days and symptoms for the Influenza virus infection. In relation to the effect of Gln supplementation in the IgA levels, in a general way, this amino acid did not show the ability to promote its elevation, especially in studies that aimed to evaluate the effect of this supplementation in the occurrence of upper respiratory tract infection (URTI) induced by acute exercise training [36,37]. It is very important to clarify that, in agreement with the literature, the production of IgA is different in the mucosa and systemic since in the mucosal immunity its composition offers the opportunity to produce the secretory IgA faster than the systemic immunity. In addition, it is well-known that a lower quantity of systemic IgA can be allocated in the mucosa [38].

Therefore, our observation that a group of elderly subjects engaged in the combined exercise training program and supplemented with Gln increased the specific-IgA levels for the Influenza vaccine both in serum and saliva could lead to putatively suggest that this combination is safe, useful and favorable to elicit a robust immune response against the Influenza virus vaccine not only in systemic but also in the mucosa.

Corroborating the remarkable effect of Gln supplementation in our study, we were able to demonstrate that the NP-Gln subgroup presented a two-fold and five-fold higher titer rate of HI for the H1N1 virus than the NP-PL subgroup and CET-subgroups, respectively. There is no doubt that the elevation in this parameter is very important, due to shows that this elderly population enhancing their capacity to neutralize the Influenza virus [39]. Although we cannot affirm, we only can suggest that the lower elevation of titer rate of HI in the CET subgroups could be impacted by the improvement of immune response both to new antigens (IgM) and also mucosal protection (IgA) observed in these subgroups.

## 5. Conclusions

Taken together, the results presented in this study can reinforce the benefits of the regular practice of combined exercise training on the specific immune response for the Influenza vaccine. In addition, the l-glutamine supplementation, in association or not with combined exercise training, was also able to improve some crucial immunological factors involved in the response for the Influenza vaccine in a population of elderly subjects.

## Figures and Tables

**Table 1:** Description of the panel used in flow cytometry to assess lymphocyte concentration, type, and profile.

Type of Cell	Profile	CD Markers	
CD4+ T cells	Activated	CD3^+^	CD4+CD38+HLA-DR+
	Naïve	CD3^+^	CD4+CD27+CD45RA+CD197+
	Effector	CD3^+^	CD4+CD27-CD45RA+CD197^-^

**Table 2:** Physical characteristics (mean and standard deviation (SD) of the NP and combined-exercise training (CET) groups supplemented with l-glutamine or placebo.

Groups Characteristics	Volunteers (*n* = 84)				
	Non-Practitioners (NP, *n* = 31)		Combined-Exercise Training (CET, *n* = 53)		*p* Value
	Placebo (*n* = 17)	l-Glutamine (*n* = 14)	Placebo (*n* = 27)	l-Glutamine (*n* = 26)	
Age (year)	75.1 ± 7.1	72.9 ± 5.4	72.2 ± 5.9	71.2 ± 5.9	>0.05
Height (m)	154.2 ± 9.5	162.3 ± 8.3 *	156 ± 9.7	156.8 ± 8.8	<0.05
Weight (kg)	66.0 ± 10.7	75.7 ± 14.5 *	61.9 ± 10.3	62.9 ± 13.0	<0.05
Body mass index (kg/m²)	27.7 ± 4.0 ^#^	28.5 ± 3.7 ^$^	25.4 ± 3.6	25.4 ± 3.9	<0.05
Total body fat (%)	39.6 ± 10.1	38.1 ± 9.1	35.4 ± 7.7	35.3 ± 7.7	>0.05
Fat-free mass (%)	60.4 ± 9.3	61.9 ± 9.2	64.6 ± 7.8	64.7 ± 7.5	>0.05
Skeletal muscle mass (kg)	18.3 ± 4.1	22.3 ± 4.0	19.6 ± 3.6	19.8 ± 3.9	>0.05
IPAQ					
Physical activity (min/week)	433.7 ± 76.1 ^#^	371.4 ± 69.2 ^$^	677.3 ± 60.4	754.1 ± 85.6	<0.05
Sitting (min/week)	1685 ± 176.5 ^#^	1874 ± 178.6 ^$^	1224 ± 112.21	1326 ± 107.74	<0.05

Note: * Different from both combined-exercise training groups. Different from the combined-exercise training placebo (^#^) and L-glutamine (^$^) groups.
